# Influence of Gestational Hormones on the Bacteria-Induced Cytokine Response in Periodontitis

**DOI:** 10.1155/2021/5834608

**Published:** 2021-10-18

**Authors:** Betsaida J. Ortiz-Sánchez, Martha Legorreta-Herrera, Miriam Rodriguez-Sosa

**Affiliations:** ^1^Dental Surgeon Career, Facultad de Estudios Superiores Iztacala, Universidad Nacional Autónoma de México (UNAM), MEX. C.P. 54090, Tlalnepantla, Mexico; ^2^Molecular Immunology Laboratory, Facultad de Estudios Superiores, Zaragoza, UNAM, Iztapalapa, Mexico City C.P. 09230, Mexico; ^3^Innate Immunity Laboratory, Facultad de Estudios Superiores Iztacala, UNAM, MEX C.P. 54090, Tlalnepantla, Mexico

## Abstract

Periodontitis is an inflammatory disease that affects the supporting structures of teeth. The presence of a bacterial biofilm initiates a destructive inflammatory process orchestrated by various inflammatory mediators, most notably proinflammatory cytokines, which are upregulated in the gingival crevicular fluid, leading to the formation of periodontal pockets. This represents a well-characterized microbial change during the transition from periodontal health to periodontitis; interestingly, the gestational condition increases the risk and severity of periodontal disease. Although the influence of periodontitis on pregnancy has been extensively reviewed, the relationship between pregnancy and the development/evolution of periodontitis has been little studied compared to the effect of periodontitis on adverse pregnancy outcomes. This review is aimed at summarizing the findings on the pregnancy-proinflammatory cytokine relationship and discussing its possible involvement in the development of periodontitis. We address (1) an overview of periodontal disease, (2) the immune response and possible involvement of proinflammatory cytokines in the development of periodontitis, (3) how bone tissue remodelling takes place with an emphasis on the involvement of the inflammatory response and metalloproteinases during periodontitis, and (4) the influence of hormonal profile during pregnancy on the development of periodontitis. Finally, we believe this review may be helpful for designing immunotherapies based on the stage of pregnancy to control the severity and pathology of periodontal disease.

## 1. Introduction

Periodontal disease is an inflammatory condition of periodontal tissues with a heterogeneous aetiology and is one of the most common diseases in the world [1]. This disease affects the gum and supporting tissues of the teeth, alveolar bone, periodontal ligament, and root cementum. Approximately 60% of the total population has some degree of periodontal disease [2]. In Latin America, this number increases to up to 90% [3]. The early stage of development of this pathology is called gingivitis and affects only the soft tissues. The severe form, called periodontitis, severely affects periodontal tissues, mainly alveolar bone, with subsequent loss of insertion of dental organs.

The development of periodontal disease and its progression depend on different factors that modulate the host immune response against the biofilm, such as genetic and epigenetic predisposition including hereditary angyoderma [4], social factors, habits (such as tobacco and alcohol use and poor oral hygiene) [5], advanced age [6], and systemic conditions, such as obesity, malnutrition [7], infections (such as HIV/AIDS), osteoporosis and stress [8], type 1 and 2 diabetes [9], and scleroderma disease [10]. Notably, periodontitis affects 23% of women between 23 and 54 years of age and is present in 56% of pregnant women [11]. Furthermore, recent evidence suggests that hormonal treatment, the use of hormonal contraceptives, and pregnancy induce clinical, cytological, or microbiological changes in women [12], which probably promote the development of this disease.

Inflammatory cytokines are upregulated during pregnancy and increased during ovulation, in early gestation, in term pregnancy, and during delivery [13]. However, it has not been established whether there is any relationship between pregnancy/proinflammatory cytokines and the development of periodontal disease. In this review, we summarize the current knowledge by providing a broad overview of periodontitis and then focusing specifically on recent findings related to the inflammatory response in pregnancy and its possible relationship to the development of periodontitis.

## 2. Periodontitis and the Oral Microbiome

The oral cavity has a dynamic environment that is formed by the oral microbiome with all of its interspecies interactions but also interactions with the oral cavity, creating a symbiotic relationship with the human host [14]. Periodontitis is initiated by polymicrobial synergy, and dysbiosis is modified by numerous risk factors. Competitive and cooperative interspecies interactions of microbial communities can shape the nature and function of the entire microbiome synergism [15]. The subgingival microbiome includes the red complex triad (*Treponema denticola*, *Tannarella Forsythia*, and *Porphyromonas gingivalis*) [16], orange complex triad (*Fusobacterium nucleatum*, *Prevotella intermedia*, and *Parvimonas micra*), *Actinobacillus actinomycetemcomitans*, *Campylobacter rectus*, *Eikenella corrodens*, *Bacteroides forsythus* [17], *Filifactor alocis* [18], *Peptoanaerobacter stomatis*, *Firmicutes phylum*, *Methanobrevibacter oralis* [19], *C. albicans* [20], and *human cytomegalovirus* and *Epstein-Barr virus* [21].

The periodontal microbiome is complex and constitutes the cornerstone in the development of periodontal disease. The characteristics of the bacteria themselves are essential in determining the course of the immune response. For example, *Porphyromonas gingivalis* can modulate the innate inflammatory response [22]. *Filifactor alocis* also induces oxidative stress and alters the recognition capacity of the inflammatory response by inactivating the complement pathways [23]. *Filifactor alocis* and other bacteria, such as *Porphyromonas gingivalis*, are highly invasive and promote dysbiosis of the microbiota [24]; therefore, a pathogenesis model has been proposed in which periodontal disease is initiated due to dysbiosis of the microbiota, called the PSD (polymicrobial synergy and dysbiosis) model [25].

## 3. Immune Response in Periodontitis

Immune cells interact with biofilms when their pattern recognition receptors (PRRs) detect pathogen-associated molecular patterns (PAMPs) present on bacteria. These receptors are expressed on innate immune cells, such as neutrophils, eosinophils, basophils, macrophages (M*φ*s), monocytes, dendritic cells (DCs), and natural killer (NK) cells, and adaptive immune cells, such as T and B lymphocytes, as well as on nonimmune cells, such as epithelial cells, endothelial cells, and fibroblasts [26]. This PAMP-PPR interaction activates the innate immune response characterized by neutrophil, eosinophil, and basophil recruitment, consequently activating the complement system [27].

**Figure 1:**
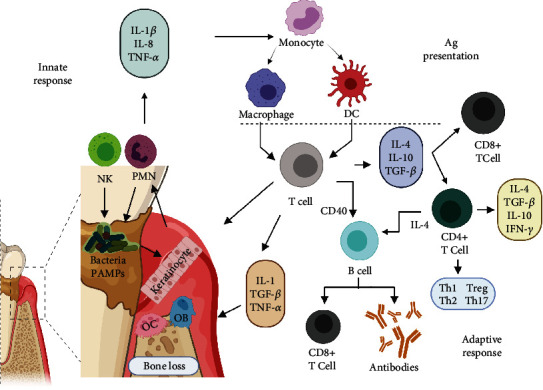
Innate immune cells, including keratinocytes, polymorphonuclear cells (PMNs), antigen-presenting cells (APCs), and natural killer (NK) cells, hold pathogen recognition receptors (PRRs) that detect pathogen-associated molecular patterns (PAMPs) present in biofilm bacteria, and these interactions promote the acute inflammatory response. Antigen-presenting cells (APCs) phagocytose pathogens, process antigens, and present the antigens in the form of peptides displayed by major histocompatibility complex (HLA) molecules. Costimulatory molecules stabilize this interaction. APCs migrate to secondary lymphoid tissues where they activate adaptive immune cells, including CD4^+^ T cells, such as Th1, Th2, and Th17 cells; cytotoxic CD8^+^ T cells; and B cells that will mature into antibody-producing plasma cells. Created with BioRender.com (https://biorender.com/).

This first recognition is characterized by acute inflammation; if biofilm dysbiosis persists, this response develops into chronic inflammation. In this phase, osteoclast activation is favoured. It results in bone resorption, with subsequent degradation of the bone matrix and periodontal ligament fibres by metalloproteinases (MMPs) and the formation of granulation tissue [28] (Figure 1). Thus, in both acute inflammation and chronic inflammation, cytokines, and inflammatory mediator's determinate disease progression factors.

Antigen-presenting cells (APCs), such as DCs, recognize pathogens expressing PAMPs, internalize these pathogens by phagocytosis, and degrade and process pathogen-derived antigens, transforming the antigens into small peptides that bind to major histocompatibility complex (HLA) molecules for display on the cell surface. This presentation is accompanied by the expression of the costimulatory molecules CD86 and CD40. DCs migrate to secondary lymphoid tissues (lymph nodes and lymphoid tissue) to present antigens and thus activate CD4^+^ T cells to generate an antigen-specific immune response [29]. CD4+ T cells differentiate into regulatory and effector T cell subsets: Th1, Th2, Th17, follicular helper T (Tfh) cells, and regulatory T cells (Tregs) [30]. The differentiation of Th1 and Th2 cells is mutually antagonistic; Treg and Th17 cells share the same origin and have opposite effects, while Th17 cells cause autoimmunity and inflammation, and Treg cells inhibit these and maintain immune homeostasis.

The activation profile of CD4^+^ T lymphocytes in periodontal disease varies depending on disease progression. In the initial phase, CD4^+^ T lymphocytes exhibit a proinflammatory Th-1 profile characterized by the synthesis of macrophage inhibitory factor (MIF), interleukin- (IL-) 2, and interferon- (IFN-) *γ*, which promote cellular immunity and the activation of cytotoxic CD8^+^ T lymphocytes (TCs) and Th-17 cells [31]. Other cytokines, such as IL-1*α*, IL-1*β*, IL-8, IL-6, and tumour necrosis factor- (TNF-) *α* produced by monocytes, M*φ*s, DCs, and neutrophils, are also produced under these conditions [32]. In addition, endothelial cells, fibroblasts, and osteoclasts produce prostaglandin E2 (PGE2) and granulocyte macrophage colony-stimulating factor (GM-CSF) [33]. Together, these conditions promote the expression of receptor activator of NF-*κ*B ligand (RANKL), leading to osteoclastogenesis [34].

In the chronic phase of periodontitis, CD4^+^ T lymphocytes differentiate towards an anti-inflammatory Th2 profile, characterized by the production of IL-4, IL-5, IL-6, and IL-10 [35]. This profile favours B lymphocyte activation and subsequent differentiation into IgG-type immunoglobulin-producing plasma cells [36]. In this way, when periodontitis becomes chronic, negative regulation of inflammation through the anti-inflammatory cytokines IL-4, IL-6, IL-10, IL-11, and IL-13 becomes predominant [37]. This immunoregulation is a complex phenomenon involving mediators such as RANKL-DCs, favouring the activation of CD4^+^ Foxp3^+^ T (Treg) cells [38], which also regulate the inflammatory Th1 response, thus, preventing the destruction of periodontal tissues [39].

Th17 cells are characterized by the production of the cytokine IL-17, although they also produce other cytokines, such as IL-17F, IL-21, IL-22, and GM-CSF [40]. Th17 cells are induced in the presence of TGF-*β*/IL-1*β*, IL-6, and IL-23 and express chemokine receptor- (CCR-) 6, which allows their migration to barrier and mucosal sites, such as gingival tissues, suggesting a protective role in the oral barrier. The inflammatory functions of Th17 cells depend on the different combinations of cytokines expressed in the local environment [33].

IL-17 signalling on epithelial cells is essential for the physiological regulation of mucosal immunity and barrier defences, promotes the production of antimicrobial factors, regulates the recruitment and generation of neutrophils through the induction of chemokines CXCL-1, 2, and 5, and induces the secretion of granulopoietic factors such as G-CSF and GM-CSF. Moreover, IL-17 induces the production of antimicrobial mediators such as *β*-defensins (HBD), regenerative proteins (ReG), S100 proteins, cathelicidins, lipocalins, and lactoferrins [40].

In periodontal lesions, an increase in Th17-related cytokines, such as IL-23 and IL-21, and other proinflammatory and osteoclastogenic mediators, such as IL-6 and RANKL, has been found [41]. IL-17 can enhance RANKL expression on osteoblasts by promoting the secretion of MMP-1, MMP-3, IL-6, and IL-8 from gingival fibroblasts and TNF release from macrophages in periodontal tissues [42] and activate RANK signalling on osteoclasts, promoting osteoclastogenesis [43]. In addition, IL-17 enhances inflammation through excessive neutrophil recruitment, enhances proinflammatory cytokine production, and activates osteoclasts, contributing to immunopathology and bone destruction.

In addition, the presence of Treg cells inhibits osteoclast formation and monocyte/M*φ* differentiation through the secretion of transforming growth factor- (TGF-) *β*, IL-4, and IL-10 and the interaction of CTLA-4 (cytotoxic T-lymphocyte antigen) with the monocyte precursor/M*φ* receptors CD80/CD86 [44].

Keratinocytes express several families of pattern recognition receptors, including TLR2, TLR4, NOD1, and NOD2, which are activated by both extracellular and intracellular bacterial molecular structures.

The mechanisms of tolerance include not only DCs but also Tregs. The function of oral Langerhans cells (LCs) under physiological conditions is to maintain a state of immune tolerance [45]. DCs also participate in peripheral tolerance in chronic periodontitis. These cells are capable of phagocytosing pathogens, but due to the anti-inflammatory cytokines IL-10 and TGF-*β*, their ability to present antigens decreases; this decrease is associated with a deficiency in the costimulatory molecules CD80 and CD86, so they cannot activate T cells properly [29]. Natural killer cells, either through direct cell-to-cell contact or indirectly through cytokines, interact with dendritic cells to mediate T cell immune responses [46].

In summary, the severity of the pathology of periodontal disease, as well as its chronicity, depends on the balance and interaction between the Th1/Th17 inflammatory response and the Th2/Treg anti-inflammatory regulatory response [47]. The Treg/Th17 balance is shifted in favour of Th17 cells in the presence of proinflammatory cytokines.

## 4. Remodelling of Bone Tissue in Periodontitis

Bone tissue is one of the most affected tissues in periodontitis; under normal conditions, it is constantly remodelled, which requires cells that degrade the bone matrix (osteoclasts) and cells that synthesize the bone matrix (osteoblasts) [48]. Briefly, the bone matrix produces the growth factors TGF-*β* and insulin-like growth factor- (IGF-) 1. Both molecules favour the recruitment of preosteoblasts and promote their maturation; subsequently, some osteoblasts differentiate into osteocytes. This mechanism is regulated by paracrine and endocrine factors, such as epinephrine B2, IL-6, and parathyroid hormone (PTH) [49].

On the other hand, osteoclasts differentiate from a myeloid precursor under the influence of M*φ*-colony-stimulating growth factor (M-CSF) and RANKL. Osteoprotegerin (OPG), produced by osteoblasts, modulates the osteoclast differentiation process [50]. Osteoclasts produce the proteolytic enzymes cathepsin K and metalloproteinases (MMPs), which degrade the bone matrix. In addition, H^+^ proton transporters and ATPase generate an acidic environment that, together with chloride channels, hydrolyses, and solubilizes both organic matter and inorganic matter. All this happens in Howship's lacunae, and the osteoclasts seal them with their podosomes [51].

In periodontitis, lymphocyte infiltrates and mononuclear cells influence and alter the homeostatic balance of the bone. Although modulation of the bone immune system is complex, the balance of proinflammatory Th1 and anti-inflammatory Th2 immune responses is critical [52]. The cytokines that promote bone resorption include IL-1*β*, TNF-*α*, IL-6, IL-15, and IL-17, and the cytokines that inhibit bone resorption include IL-4, IL-10, IL-13, IL-18, GM-CSF, and IFN-*γ* [50]. The best example is TNF-*α*, which activates osteoclasts and inhibits osteoblast differentiation with a consequent decrease in bone formation [53]. Specifically, TRAIL (TNF-related apoptosis-inducing ligand) participates in osteoblast apoptosis and low bone quality in periodontitis [54].

The other important factor in bone resorption is a member of the TNF family, RANKL, which promotes osteoclast differentiation and modifies the relationship between osteoblasts and osteoclasts [55]. RANKL is overexpressed in proinflammatory systems, and the major source is B lymphocytes, followed by T lymphocytes and finally monocytes, although osteoblasts also produce RANKL after activation through TLRs [56].

The RANKL-RANK-OPG system is involved in bone regulation via regulation of the immune system to control other systems and several pathologies. These interactions have been described mainly in rheumatoid arthritis, which involves bone loss and bone remodelling [57, 58]. The regulation of the RANKL-RANK-OPG system and its mechanisms should be clarified in periodontitis since modulation of these mechanisms may favour treatment and prevent disease sequelae.

### 4.1. Involvement of Metalloproteinases in Bone Remodelling

Periodontal tissues are composed of connective tissue; the extracellular matrix (ECM) is mainly formed by collagen types I, III, IV, V, and VI and noncollagenous proteins, including elastin, fibronectin, laminins, and proteoglycans. In periodontitis, significant degradation of all of these constituent elements of periodontal tissues occurs. Overexpression of MMPs, a family of zinc-dependent endopeptidases, is associated with the development and severity of periodontal structure loss in this pathology. These enzymes are capable of degrading most of the components of the ECM [59]. In addition, MMPs favour processes involved in inflammation, such as inflammatory cell migration, chemokine recruitment and processing, cleavage and neutralization of complement components, phagocytosis, and cell lysis [60].

Different MMPs have the specificity to act in the degradation of specific types of tissues, e.g., MMP-2 in its proenzyme form (~72 kDa) and in its active form (~59–62 kDa) and the MMP-9 proenzyme (~92 kDa) and active forms (~88 kDa) degrade fibronectin, elastin, and collagen types IV, V, VII, X, XI, and XII; in acidic medium, they can degrade collagen type I [61]. MMP-13, both in its proenzyme form (~60 kDa) and in its active form (~45–50 kDa), degrades collagen I, collagen II, collagen III, collagen IV basal membrane, proteoglycans, fibronectin, fibrin, and tenasin [62].

The mechanisms that regulate homeostasis, such as the overexpression of MMPs in periodontitis, are complex processes. In homeostasis, one of the regulatory pathways involves tissue inhibitors of MMPs (TIMPS) and *α*2-macroglobulins that bind covalently and irreversibly to the active site of MMPs with high affinity. The levels of these endogenous mediators are elevated in healthy tissue and various fluids, such as serum, amniotic fluid, and saliva, and these mediators are synthesized by fibroblasts, monocytes, M*φ*s, endothelial cells, and osteoblasts [63]. Another mechanism of negative regulation of MMP expression is the presence of the oestrogen 17*β*-oestradiol, which negatively regulates the flow of calcium into cells [64] and consequently reduces the expression of MMPs, particularly MMP-1 [65].

On the other hand, the overexpression of MMPs can be triggered by different factors, such as the presence of PGE_2_ [66], *in vitro* and *in vivo* are influenced by mechanical load as orthodontic movement [67, 68], interactions with periodontopathogenic bacteria, such as *Eikenella corrodens* [69], *Porphyromonas gingivalis*, and *Prevotella intermedia* [70], or polysaccharides and cytokines, such as IL-1*β* and TNF-*α*. In any case, these factors act on monocytes and M*φ*s by favouring the production of mediators that function as activators or modulators of MMPs [71]. For example, MMP-13 upregulates RANKL/OPG levels by activating MMP-9, increases TGF-*β* signalling in metastatic bone lesions [72], and influences osteoclastic activity [73].

Undoubtedly, the participation of MMPs in the development and severity of periodontitis is known. Establishing whether their expression is affected by hormonal conditions, such as gestation, is important because regulating their expression could be considered a component of the therapeutic treatment of gestational periodontitis.

## 5. Sex Hormones and Periodontitis

Sex hormones modulate immune functions, such as thymocyte maturation and selection, cell migration, MHC-II expression, cell proliferation, and cytokine production (Table 1) [74].

The regulatory effects exerted by hormones on the immune response depend on interactions with their receptors. For example, B cells have high expression of the genes encoding the two oestrogen receptors, ER1 and ER2. There is a moderate expression of these receptors on CD4^+^ T, CD8^+^ T, NK, and plasmacytoid DCs, while monocytes express reduced levels of ER1. Estradiol and ERs bind to transcription factors, such as NF*κ*B, SP1, AP-1, and C/EBP*β*, that are involved in the regulation of different cellular functions [99].

Progesterone receptors (PRs) are present on epithelial cells, mast cells, eosinophils, NK cells, M*φ*s, plasmacytoid DCs, and CD4^+^ and CD8^+^ T lymphocytes. Interestingly, the expression of PRs is higher in DCs from female rats than in those from male rats [100], which makes it clear that the expression of these receptors and consequently the response that is generated when their ligand binds are higher in females. Different PRs include two intracellular receptors (iPRs) and three membrane receptors (mPRs), with two isoforms each. iPRs were initially described in the lymphocytes of pregnant women, while mPRs were described in T lymphocytes and are overexpressed during the luteal phase in CD8^+^ T lymphocytes. Differential expression of PRs may partially explain the differential activation of immune cells and differences in susceptibility to various infectious and noninfectious diseases between men and women [101].

In the same context, the sex hormone profile also has an impact on subgingival microbiology. It has been demonstrated that this profile promotes the development of periodontopathogenic bacteria, such as *Porphyromonas gingivalis* [102], subgingival anaerobic-aerobic bacteria, *Prevotella melaninogenica*, and *Prevotella intermedia* [103]. It is widely recognized that hormones related to gestation alter the immune response, modifying the pathogenesis of some diseases; for example, in multiple sclerosis and autoimmune encephalomyelitis, where an exacerbated inflammatory response is associated with the severity of the pathology, the disease severity decreases during gestation. Diseases such as malaria and influenza, which require acute inflammatory responses for their control, are exacerbated during pregnancy [80]. This phenomenon could be associated with estriol concentrations, which increase significantly during gestation. Estriol is produced in high concentrations by the fetoplacental unit during pregnancy; it accounts for 90% of the oestrogen produced during pregnancy, while the other 10% corresponds to oestradiol [104]. Although the immunological functions of estriol are similar to those of oestradiol because they share receptors, estriol seems to differentially influence the immune response; in experimental models of autoimmune pathologies, when estriol was administered, decreases in the proinflammatory cytokines TNF-*α* and IFN-*γ* have been observed, in addition to decreases in CD4^+^ and CD8^+^ cells [105]. This immune response modified by the presence of estriol, together with other hormones present during pregnancy, could also influence the development of periodontitis. This observation is corroborated in pregnant women, who, due to their condition, have a modified hormonal profile that consequently favours the accumulation of *Bacteroides*, which is increased in abundance up to 55 times in pregnant women compared to nonpregnant women [106].

The involvement of hormones other than estriol in the development of periodontal diseases has been widely documented. Progesterone increases vascular permeability and favours oedema, erythema, and gingival bleeding, which are all associated with increased populations of *Porphyromonas gingivalis*, *Prevotella intermedia* [107], *Actinobacillus actinomycetemcomitans* [108], and *Prevotella melaninogenica* [109].

Oestrogens, particularly oestradiol, favour angiogenesis and fibroblast proliferation and promote osteoblast differentiation and maturation, osteoprotegerin (OPG) and RANKL expression in osteoblasts, and osteoclast apoptosis by inhibiting osteoclast activity [83]. Periodontal ligament (PLD) cells synthesize RANKL and OPG. *In vitro* cultures of oestrogen-treated PLD cells increase OPG expression and decrease RANKL expression through ER2 [110]; these observations demonstrate that oestrogens can modulate the activity of periodontal tissues and promote homeostasis.

Androgens participate in bone growth; they are anabolic agents that increase bone mass, mainly in males, although different androgens, including testosterone, are also present in females. Androgen receptor mRNA is expressed more in cortical osteoblasts than in trabecular bone and is more closely related to cortical osteoblasts, which generate a thicker cortical bone layer in males, while in osteoblasts, androgen receptor mRNA is expressed similarly between the sexes. Androgens promote osteoblast differentiation and decrease osteoclast apoptosis; specifically, dihydrotestosterone reduces OPG levels. The functions of androgens in women have not been clearly defined; however, they are involved in the maintenance of bone density [100].

Sex hormones are involved in bone regulation and immune system maturation and modulate the function of nonsexual tissues; therefore, these hormones may play a central role in the development of periodontitis in different stages of life.

### 5.1. Pregnancy and Periodontitis

Gestation is a condition that involves physiological changes in the mother, and these changes should allow “immune tolerance” towards the foetus to develop, as well as the appearance of new cells, such as trophoblasts [111].

Recently, the relevance of the model of a foetus as a semiallograft capable of inducing the absence of a specific immune response to prevent its destruction has been debated. It is not a simple absence of the immune response but a state of immunoregulation that allows the implantation of a foetus, which is also able to respond to injury or aggression from the environment with an immune response endowed with specificity and memory [112]. This immune tolerance, in order to not reject the foetus and at the same time allow protection of pregnant woman against pathogens, requires the transient modification of immunity, which favours a Th2 environment over a Th1 environment [113]. Different mechanisms have been described to explain immune tolerance to paternal antigens, including tolerance induction in T lymphocytes, including Treg and Th17 cells [114]. In a healthy pregnancy, the Th17/Treg ratio shifts in favour of Treg cells, while a decrease in Treg cells or an increase in Th17 cells is detrimental to normal pregnancy [115]. Tolerance is promoted by Treg and Th2 cells by repressing Th1 and Th17 cells, while Th17 cells protect trophoblasts from pathogens [116].

**Figure 2:**
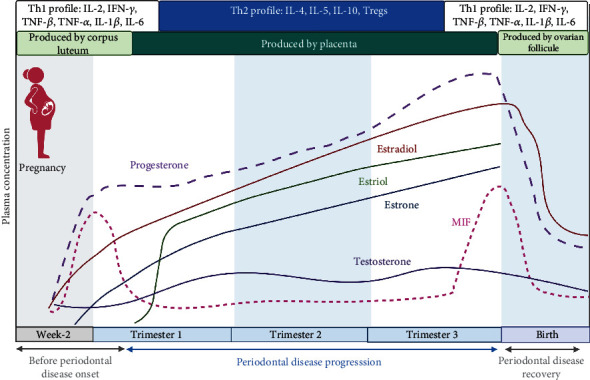
Time course of pregnancy hormones, periodontal disease, and the proinflammatory cytokine MIF. During early gestation, hormones are produced in the corpus luteum, and later until the end of gestation, they are produced in the placenta. Estriol is also synthesized in the placenta, and its production ceases when the pregnancy reaches term. Therefore, embryo implantation and early gestation require a Th1 profile with the expression of proinflammatory cytokines. During the second half of gestation, the expression of these cytokines decreases, and the profile is a Th2 and Treg cell profile, which is maintained until delivery, when the profile returns to a Th1 profile. The expression of MIF, which is a proinflammatory cytokine, maintains this trend during pregnancy. Created with BioRender.com (https://biorender.com/).

During gestation, the maternal-foetal interaction and the development of the placenta favour increased hormone concentrations. In particular, the placenta synthesizes and releases oestrogen and progesterone into circulation. This initiates events that stimulate “suppressive” immune responses, mainly at the level of lymphocytes. Suppression of CD4^+^ and CD8^+^ T lymphocytes decreases the secretion of IL-2, IFN-*γ*, TNF-*β*, TNF-*α*, IL-1*β*, and IL-6 [117]. The levels of oestradiol in human serum are ~0.1 *μ*M, and those in blood from the intervillous space are ~0.25 *μ*M, which is ~25 times higher than the concentration found in nonpregnant women at the midovarian cycle stage [118]. Oestradiol at a concentration of 0.04 ng/mL or higher and progesterone at 0.1 ng/mL both inhibit lipopolysaccharide- (LPS-) induced IL-1 and TNF-*α* secretion in monocyte cultures. In addition, the switch from the Th1 profile to the Th2 profile and the suppression of the cytolytic function of NK cells are processes regulated by progesterone-induced blocking factor (PIBF), which is secreted by CD8^+^*γδ* T cells [119]. High concentrations of PIBF favour the differentiation of CD4^+^ T cells into Th2 lymphocytes, which increases IL-4, IL-5, and IL-10 concentrations and promotes the prevalence of an anti-inflammatory profile (Figure 2) [120].

In the gestational stage, polymorphonuclear cells (PMNs) show decreased chemotaxis and adhesion beginning in the second trimester and continuing throughout gestation [121]. This altered neutrophil activation and depressed leukocyte function during pregnancy may explain susceptibility to certain infections [122]. For example, gingival inflammation has been associated with increased serum levels of oestrogen and progesterone, even though no changes in TNF-*α* or IL-1*β* levels have been detected [123].

In animal models, levels of the cytokines IL-1*β*, IL-6, IL-8, IL-17, and TNF-*α* increase under different conditions. For example, in maternal infections with periodontal pathogens or *in vitro* models of placental cells and tissues, exposure to periodontal bacteria or products induces the secretion of COX-2, IL-8, IFN-*γ*, and TNF-*α* in addition to causing apoptosis [124], and PGE_2_ causes uterine contractions [125].

On the other hand, during pregnancy, periodontal alterations increase, and the ratio of anaerobic to aerobic bacteria is modified in the second trimester of pregnancy, mainly through increases in *Prevotella melaninogenica* and *Prevotella intermedia* related to the plasma concentrations of estrogens and progesterone [126]. In animal models of periodontitis established with *Porphyromonas gingivalis* in pregnant mice, an increased immune response with decreased expression of anti-inflammatory cytokines and increased destruction of periodontal tissues is observed [102], and an imbalance in the Th17/Treg cell ratio with aggravation of periodontitis during pregnancy occurs [127].

Periodontitis causes gynecological problems, ranging from difficulty in embryo implantation to preterm delivery and low birth weight. Two possible causes have been proposed: first, periodontal bacteria cause infections in the placenta and foetus; second, inflammation can provoke responses at the maternal-foetal interface [128]. There are several contrasting and inconclusive reports on patients with recurrent miscarriages and multiple implantation failures during *in vitro* fertilization cycles which have a prevalent Th1 profile in their peripheral blood lymphocytes [129].

Offenbacher et al. noted that primary infections in distant systems can guide a pregnancy to an abnormal term [130]. Periodontal disease is an infectious process in periodontal tissues characterized by an increase in proinflammatory cytokines, and during pregnancy, the concentration of prostaglandins increases [35]. Therefore, there may be a relationship between both factors; periodontitis influences pregnancy, and that gestation influences the severity of periodontitis. One of the possible causes is the spread of bacteria or inflammatory mediators of periodontal origin by different routes, including (1) bacterial blood spread (bacteremia), (2) blood dissemination of inflammatory mediators, and (3) transmission of oral pathogens and colonization of the vaginal microbiome [131]. González-Jaranay et al. reported that in pregnant women with some degree of periodontitis, symptoms progress and worsen throughout gestation. However, in the postpartum period, clinical data improve [132]. Other authors have noted that maternal periodontal disease is not a risk factor if infectious processes are controlled [133].

Regarding the interaction of gestation with periodontitis, some studies did not find strong evidence of this interaction; however, they proposed routine periodontal therapy in pregnant women as a safe treatment for mothers and foetuses, in addition to improving the clinical signs of maternal periodontal disease [134]. In contrast, evidence of strong links between periodontitis and pregnancy disorders such as preeclampsia, preterm delivery, and low birth weight, attributable to periodontal disease, has recently been reported [135], as have associations of periodontitis with metabolic disorders such as obesity and diabetes [136].

## 6. Conclusions

This review shows that sex hormones modulate the immune response and participate in processes such as the maturation and selection of immune cells, cell trafficking, expression of histocompatibility molecules, cell proliferation, and cytokine production. Although pregnancy is a condition that modifies the hormonal profile, little is known about its effects on the development of periodontitis. Here, we collect important evidence that gestational hormones, such as 17*β* oestradiol, estriol, and progesterone, influence the development of periodontitis. Importantly, the interaction between the concentration of gestational hormones and periodontal disease appears to be bidirectional: on the one hand, the hormonal profile during pregnancy seems to be decisive for the development and severity of periodontal disease, but on the other hand, the infectious process associated with periodontitis during pregnancy generates a proinflammatory immune profile that can produce alterations such as preeclampsia, preterm delivery, and low birth weight.

However, future studies are needed to understand the immune mechanisms underlying the interaction of pregnancy and periodontal diseases. The information gathered here has the potential to contribute to an understanding of the role of hormones in the development of periodontitis, allowing dental teams that care for pregnant and childbearing women to develop preventive and therapeutic strategies.

## Figures and Tables

**Table 1:** Influence of sex hormones on some cells and cytokines of the immune response.

Hormone	Regulation	References
17*β*-estradiol	↑TCD8+ from spleen and in vitro. ↑ maturation and activation of B lymphocytes, ↓Ig2a in peripheral blood mononuclear cells and spleen cells. ↓TNF-*α*, ↑IFN-*γ* e ↑IL-10 in peripheral blood mononuclear cells, spleen, and in vitro. Peritoneal M*φ* ↑, TLR4 in vitro. ↑DCs ↑IL-12 in bone marrow, in vivo. Inhibits apoptosis by TNF-*α* via PI3k/Akt in neural progenitor cells. ↓IL-1*β* and TNF-*α* in bone marrow. [↓E2] ↑Th1, [↑E2] ↑Th2 in peripheral blood mononuclear cells in vitro.	[75] [76, 77] [78, 79] [80] [81] [82] [83] [84]

Progesterone	↓M*φ*, DCs, and NKs in peripheral blood mononuclear cells ↓NF*κ*B transduction. ↑Th2, ↑IL-4 e ↑IL-5, ↑Tregs, and ↓TH17 in peripheral blood mononuclear cells.	[85] [86] [87, 88]

Testosterona	↓LB, ↑apoptosis in bone marrow and lymph nodes. ↑TCD8+ in peripheral blood mononuclear cells. ↑M*φ*, ↑TNF-*α*, ↑CCR2, ↑[IL-10], and ↓IFN-*γ* in skin and spleen. M*φ*, ↑IL-12 e ↑IL-1*β* in vitro. DCs ↓TNF-*α*, nitric oxide, TLR-4. ↓IgG e IgM, peripheral blood mononuclear cells. ↑TGF-*β* e ↑IGFs ↑bone apposition, ↓ IL-6 osteoclastogenesis.	[89] [90] [91, 92] [93] [94, 95] [96] [97, 98]

## Data Availability

No data were used to support this study.
